# Knowledge, attitudes, and organ donor registration among medical and health sciences students: A cross-sectional study in Ras Al Khaimah, UAE

**DOI:** 10.1371/journal.pone.0362203

**Published:** 2026-11-03

**Authors:** Rasha Aziz Attia Salama, Naveen Kumar, Mohamedanas Patni, Shadha Nasser Mohammed Bahutair, Sufyan Mohammed, Saiha Khan, Minha Shehed, Omar Khalid

**Affiliations:** 1 Department of Community and Family Medicine, College of Medicine, Ras Al Khaimah Medical and Health Science University, Ras Al Khaimah, United Arab Emirates; 2 Department of Anatomy, College of Medicine, Ras Al Khaimah Medical and Health Science University, Ras Al Khaimah, United Arab Emirates; 3 Department of Obstetrics and Gynecology, College of Medicine, Ras Al Khaimah Medical and Health Science University, Ras Al Khaimah, United Arab Emirates; 4 College of Medicine, Ras Al Khaimah Medical and Health Science University, Ras Al Khaimah, United Arab Emirates; Sultan Qaboos University College of Medicine and Health Science, OMAN

## Abstract

**Background:**

Healthcare students serve as frontline ambassadors in promoting public awareness and shaping attitudes toward organ donation. This study assessed knowledge, attitudes, and organ donor registration among university students in Ras Al Khaimah, UAE, and identified factors associated with organ donor registration.

**Methods:**

A cross-sectional study was conducted among undergraduate health sciences students using a structured self-administered questionnaire to collect data on sociodemographic characteristics, knowledge of organ donation and transplantation, attitudes, and donor registration status.

**Results:**

The study included 428 students. The mean knowledge score was 7.77 ± 2.85 out of 19, indicating gaps in understanding of the definition of brain death, donor eligibility, and legal requirements. The mean attitude score was 41.58 ± 6.61 out of 65, suggesting generally positive attitudes towards organ donation, although concerns regarding organ misuse, body disfigurement, lack of trust, and fear of reduced quality of medical care were reported. Only 28.0% of participants were registered as organ donors, with a lack of awareness (39.0%) being the most frequently reported barrier among non-registered students. In multivariable logistic regression analysis, non-Arab students had lower adjusted odds of registration than Arab students, including Emiratis (aOR = 0.149, 95% CI: 0.072–0.310, p < 0.001). Compared with medical students, pharmacy (aOR = 0.050), dentistry (aOR = 0.032), and nursing students (aOR = 0.024) had significantly lower adjusted odds of registration (all p < 0.001). Students with a medical parental educational background also had lower adjusted odds of registration than those with a non-medical parental background (aOR = 0.129, 95% CI: 0.063–0.264, p < 0.001). Knowledge and attitude scores were not significantly associated with registration.

**Conclusion:**

Despite generally positive reported attitudes toward organ donation, self-reported donor registration remained low, highlighting a discrepancy between stated willingness and registration behavior. Educational and awareness initiatives should address knowledge gaps, misconceptions, trust-related concerns, and perceived barriers to registration.

## Introduction

Organ transplantation is one of the most effective life-saving interventions for patients with end-stage organ failure and is associated with substantial improvements in survival and quality of life. However, despite advances in transplantation medicine, the demand for transplantable organs continues to exceed supply worldwide. Recent global data indicate that although more than 150,000 solid organ transplants are performed annually, this represents only a fraction of the actual need, leaving many patients on waiting lists with significant morbidity and mortality [1]. Organ donation and transplantation rates vary considerably between countries and may be influenced by national consent systems and other contextual factors [2].

An individual's decision to donate organs is a complex interplay between clinical knowledge, cultural values, religious beliefs, and confidence in the health care system. According to the World Health Organization, ethical and sustainable transplantation systems must be grounded in voluntary donation, transparency, and robust public engagement [3]. Previous research has described differences between expressed willingness to donate and donor registration, although such differences do not, by themselves, establish how intentions change into behavior over time [4]. Health care students represent a key group in organ donation education and advocacy. As future health care providers, they may contribute to patient education, public awareness, and advocacy for organ donation.

Studies among university students have reported variation in organ donation knowledge and attitudes, including differences across demographic groups [5,6]. These findings highlight the importance of organ donation education among future healthcare professionals.

In the UAE, following the launch of the Hayat national program by the Ministry of Health and Prevention, organ donation and donor registration have received increasing attention [7]. Sociocultural and informational barriers to organ donation have also been reported [8]. Given the country's multicultural and diverse population, examining these factors among healthcare students may help inform culturally appropriate educational interventions. Previous research in the UAE and elsewhere has largely focused on knowledge, attitudes, and willingness toward organ donation, whereas evidence regarding actual donor registration behavior remains limited [8]. This distinction is important because stated willingness and completed registration represent different outcomes, and the factors associated with any observed difference require further investigation. Therefore, this study aimed to assess the knowledge, attitudes, and donor registration practices among students at Ras Al Khaimah Medical and Health Sciences University (RAKMHSU), UAE, and to identify factors associated with donor registration.

## Materials and methods

### Study design and setting

A cross-sectional study was conducted at RAKMHSU, United Arab Emirates, from December 1, 2024, to April 2025. As a multidisciplinary institution, RAKMHSU comprises four colleges: Medicine, Dentistry, Pharmacy, and Nursing, offering a diverse academic and sociocultural environment. The study was reported in accordance with the STROBE statement for cross-sectional studies.

### Study population and sampling

The study population included all undergraduate students enrolled at RAKMHSU during the study period, with an estimated total population of approximately 1,500 students. Students from all academic years, genders, and nationalities were eligible to participate. The minimum required sample size (n = 306) was calculated using a single population proportion formula with a 95% confidence level, 5% margin of error, and an assumed prevalence of 50% to maximize sample size in the absence of prior estimates [9]. Eligible participants included students aged 18 years or older who provided informed consent. Recruitment continued during the study period until the required sample size was met. A consecutive non-probability sampling technique was used to recruit students across all four colleges and various academic years. This approach was chosen for its feasibility within the academic setting; however, its potential for selection bias and limited representativeness was acknowledged.

### Data collection instrument

Data were collected using an anonymous, structured, self-administered questionnaire adapted from previously published questionnaires [10–12]. The questionnaire was tailored to the UAE context, incorporating items specific to national legislation and the Hayat organ donation program. It consists of four parts: sociodemographic characteristics; knowledge of organ donation and transplantation; attitudes toward organ donation; and self-reported organ donor registration. The knowledge was assessed using 19 items, with responses scored as 1 for correct and 0 for incorrect/don't know answers. The total scores range from 0 to 19.

Attitudes towards organ donation were assessed using 13 items rated on a 5-point Likert scale, with responses coded as 5 (strongly agree), 4 (agree), 3 (neutral), 2 (disagree), and 1 (strongly disagree). Item scores were summed to obtain an overall attitude score ranging from 13 to 65, with higher scores indicating greater agreement with the attitude statements.

Content validity of the adapted questionnaire was assessed through expert review by three faculty members in Community Medicine and Public Health, who evaluated the relevance, clarity, and appropriateness of the items to the UAE context. A pilot study involving 15 students from the target population was conducted to assess the clarity, feasibility, and internal consistency of the questionnaire. These participants were excluded from the final study sample (N = 428). Cronbach’s alpha was 0.70 for the 19-item knowledge scale and 0.70 for the 13-item attitude scale, indicating acceptable internal consistency for both scales.

### Data collection procedure

The questionnaire was distributed in person by the research investigators in classrooms and common student areas. Participants were provided with a brief overview of the study objectives and requested to provide written informed consent before participation. The questionnaire took approximately 10–15 minutes to complete. Following the submission of the survey, participants received an educational pamphlet designed to enhance awareness of organ donation and transplantation. Although the impact of this material was not evaluated as a study outcome, it provided essential information on the importance of donation, types of donated organs and tissues, and common misconceptions. The pamphlet also outlined the Islamic perspectives on donation and described the eligibility criteria and registration procedures of the UAE Hayat program.

### Statistical analysis

Data were initially organized and cleaned in Microsoft Excel before being analyzed using IBM SPSS Statistics version 23.0 (IBM Corp., Armonk, NY, USA). Descriptive statistics were reported as frequencies and percentages for categorical variables, and as means with standard deviations for continuous variables. Knowledge and attitude scores were treated as continuous variables following an inspection of their distributions. To compare mean scores across demographic groups, independent-sample t-tests and one-way analysis of variance (ANOVA) were used as appropriate. Before inferential testing, parametric assumptions, including normality and homogeneity of variance, were verified. Where ANOVA showed statistically significant results, post hoc comparisons were conducted using Tukey's Honestly Significant Difference test, with only significant pairwise differences reported. Finally, a multivariable logistic regression analysis was conducted to examine factors independently associated with organ donor registration, defined as a binary outcome (registered versus not registered). The model included age, gender, nationality, college, year of study, residence, parental education level, parental educational background, knowledge score, and attitude score as explanatory variables. Nationality was dichotomized into Arab (including Emirati) and non-Arab participants. Parental education level was categorized as low (no formal or primary education) and high (secondary education or above). The reference categories were male gender, Arab nationality (including Emirati), Medicine college, fifth year of study, low parental education level, non-medical parental educational background, and residence in Ras Al Khaimah. Age, knowledge score, and attitude score were entered as continuous variables. The analyses were reported as adjusted odds ratios (aORs) with 95% confidence intervals (CIs). Overall model performance was further assessed using the likelihood-ratio omnibus test, the Hosmer-Lemeshow goodness-of-fit test, Cox and Snell R-squared, Nagelkerke R-squared, and classification accuracy. A two-tailed p-value < 0.05 was considered statistically significant.

### Ethical considerations

Ethical approval was obtained from the University Human Ethics Committee of Ras Al Khaimah Medical and Health Sciences University (Approval No.: RAKMHS-HEC-143-UG-M). The research was carried out in accordance with the principles of the Declaration of Helsinki. Written informed consent was obtained from all participants before their inclusion in the study. Participation was voluntary, and participants were informed about their right to withdraw from the study at any time. Non-identifiable coded questionnaires were used to maintain anonymity. All the collected data were treated as confidential and used solely for this research.

## Results

The study included 428 undergraduate students, the majority of whom were female (65.7%, n = 281) and aged between 18 and 20 years (70.8%, n = 303), with a mean age of 19.76 ± 1.59 years. Most participants were of Arab nationality (46.3%, n = 198) and resided in emirates other than Ras Al Khaimah (69.2%, n = 296). Students from the College of Medicine constituted the largest proportion (42.3%, n = 181), and the majority were first- and second-year students, each accounting for 30.4% (n = 130). Additionally, most participants reported having parents with a high level of education (84.8%, n = 363) and a non-medical educational background (64.7%, n = 277) (Table 1).

**Table 1:** Sociodemographic characteristics of participants (N = 428).

Variable	Category	n (%)
Age (years)	18–20 21–25 26–30	303 (70.8) 122 (28.5) 3 (0.7)
Mean ± SD	19.76 ± 1.59	
Gender	Male Female	147 (34.3) 281 (65.7)
Nationality	Emirati Arab Non-Arab	104 (24.3) 198 (46.3) 126 (29.4)
Place of Residence	Ras Al Khaimah Other Emirates	132 (30.8)296 (69.2)
College	Medicine Dentistry Nursing Pharmacy	181 (42.3) 91 (21.3) 83 (19.3) 73 (17.1)
Year of Study	1st 2nd 3rd 4th 5th	130 (30.4) 130 (30.4) 87 (20.3) 61 (14.2) 20 (4.7)
Parental Education Level	Low High	65 (15.2) 363 (84.8)
Parental Educational Background	Medical Non-medical	151 (35.3) 277 (64.7)

### Knowledge of organ donation

The overall mean knowledge score was 7.77 ± 2.85 out of 19, corresponding to 40.9% of the maximum possible score. More than half of the participants were unaware of the national organ donation program (Hayat) (55.2%, n = 236). Among those aware, social media was the most reported source of information (28.5%), whereas organized awareness campaigns were the least commonly cited source (16.6%) (Fig 1).

**Fig 1:**
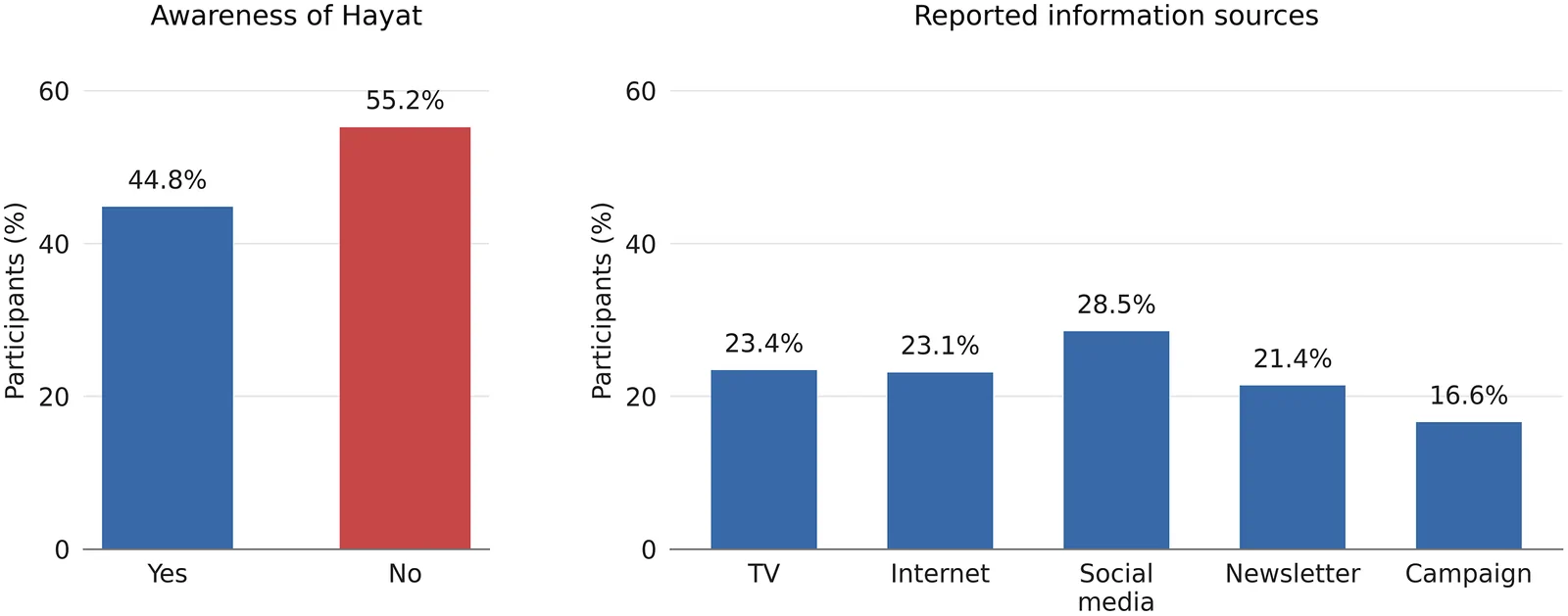
Awareness of the Hayat program and sources of knowledge among the studied participants.

Knowledge of brain death was limited, with only 48.6% (n = 208) correctly identifying its definition. Knowledge of the legal requirements for organ donation in the UAE was limited, with only 52.1% (n = 223) of respondents being aware that donation requires prior consent or family approval. Misconceptions about donor eligibility were also evident, as only 46.7% (n = 200) correctly identified that individuals with certain medical conditions were still eligible for donation. In addition, awareness of the existing infrastructure was suboptimal; 44.9% (n = 192) of the students reported familiarity with existing organ donation facilities and services in the UAE.

Knowledge score differed significantly according to gender, nationality, college, emirate of residence, and parental education level (Table 2). Females had a higher mean knowledge score than males (7.99 ± 3.03 vs. 7.35 ± 2.42, p = 0.027). Knowledge scores differed significantly across nationality groups (p = 0.045), with non-Arab students having the highest mean score (8.02 ± 3.08), followed by Arab (7.93 ± 2.75) and Emirati students (7.17 ± 2.68). However, Tukey HSD post-hoc analysis showed no statistically significant pairwise differences between nationality groups. Significant differences were also observed across colleges (p < 0.001), with medical students having the highest mean knowledge score (9.04 ± 2.72), followed by nursing students (8.27 ± 2.94), while dentistry (6.55 ± 2.02) and pharmacy students (5.59 ± 1.88) had lower mean scores. Tukey HSD post-hoc analysis showed that medical students had significantly higher knowledge scores than dentistry and pharmacy students, while nursing students had significantly higher scores than dentistry and pharmacy students (all p < 0.001). Students residing in Ras Al Khaimah had a higher mean knowledge score than those residing in other emirates (8.21 ± 2.72 vs. 7.58 ± 2.89, p = 0.033). Knowledge scores also differed significantly according to parental education level, with students whose parents had a high education level having a higher mean score than those whose parents had a low education level (7.94 ± 2.82 vs. 6.86 ± 2.86, p = 0.005). In contrast, no statistically significant differences in knowledge scores were observed according to age (p = 0.395), year of study (p = 0.298), or parental educational background (p = 0.120) (Table 2).

**Table 2:** Comparison of mean knowledge scores on organ donation and transplantation across sociodemographic characteristics of the participants.

Variable	Mean ± SD	P value
Age (years) 18–20 21–25 26–30	7.86 ± 2.9 27.52 ± 2.65 9.00 ± 2.65	0.395 a
**Gender** Male Female	7.35 ± 2.42 7.99 ± 3.03	0.027 b*
**Nationality** Emirati Arab Non-Arab	7.17 ± 2.68 7.93 ± 2.75 8.02 ± 3.08	0.045 a*
**College** Medicine Pharmacy Dentistry Nursing	9.04 ± 2.72^C^ 5.59 ± 1.88^d^ 6.55 ± 2.02^d^ 8.27 ± 2.94^c^	<0.001 a*
**Year of Study** 1st 2nd 3rd 4th 5^th^	8.14 ± 2.74 7.60 ± 2.84 7.91 ± 3.21 7.36 ± 2.42 7.20 ± 3.11	0.298 a
**Emirate of Residence** Ras Al Khaimah Other Emirates	8.21 ± 2.72 7.58 ± 2.89	0.033 b*
**Parental Education Level** Low High	6.86 ± 2.86 7.94 ± 2.82	0.005 b*
**Parental Educational Background** Medical Non-medical	7.48 ± 2.48 7.93 ± 3.02	0.120 b

^a^**P-value of ANOVA; significant ANOVA results were followed by Tukey HSD post-hoc analysis.** ^b^P-value of an independent t-test. ^c,d^For Tukey HSD comparisons, groups with different superscript letters are significantly different, whereas groups with the same superscript letter are not significantly different (p < 0.05). *P < 0.05 is statistically significant.

### Attitudes toward organ donation

The mean attitude score was 41.58 ± 6.61 out of 65. Key concerns included fear-related attitudes, with 41.8% (n = 179) of participants reporting that concerns about body disfigurement could influence their decision. Additionally, 38.6% (n = 165) expressed concern regarding the misuse of donated organs. Trust-related concerns were also reported. Approximately 34.1% (n = 146) of participants reported a lack of trust in the organ donation system. In comparison, 29.7% (n = 127) believed that registering as an organ donor might negatively affect the quality of medical care they receive. Finally, ethical uncertainty was observed, with 36.2% (n = 155) of participants expressing neutral or negative views toward presumed consent policies. Attitude scores differed significantly according to age, college, year of study, emirate of residence, and parental educational background. Mean attitude scores differed significantly across age groups (p = 0.033), with students aged 18–20 years having the highest mean score (42.11 ± 6.49), followed by those aged 26–30 years (42.00 ± 2.65) and 21–25 years (40.26 ± 6.82). Tukey HSD post-hoc analysis showed that students aged 18–20 years had significantly higher attitude scores than those aged 21–25 years (p = 0.024). Significant differences were also observed across colleges (p < 0.001), with nursing students having the highest mean attitude score (45.07 ± 6.87), followed by medical students (43.94 ± 5.85), while dentistry (37.95 ± 5.61) and pharmacy students (36.32 ± 2.80) had lower mean scores. Tukey HSD post-hoc analysis showed that medical and nursing students had significantly higher attitude scores than dentistry and pharmacy students (all p < 0.001). Attitude scores also differed significantly by year of study (p = 0.019), with first-year students having the highest mean score (42.75 ± 6.90) and fourth-year students the lowest (39.41 ± 6.03). Tukey HSD post-hoc analysis showed that first-year students had significantly higher attitude scores than fourth-year students (p = 0.010). Students residing in Ras Al Khaimah had a significantly higher mean attitude score than those residing in other emirates (42.81 ± 7.63 vs. 41.04 ± 6.04, p = 0.010). Students with a non-medical parental educational background also had a significantly higher mean attitude score than those with a medical parental background (42.25 ± 6.84 vs. 40.36 ± 6.00, p = 0.004). In contrast, attitude scores did not differ significantly according to gender (p = 0.635), nationality (p = 0.840), or parental education level (p = 0.761) (Table 3).

**Table 3:** Comparison of mean attitude scores (out of 65) according to sociodemographic characteristics (N = 428).

Variable	Mean ± SD	P value
**Age (years)**		
18–20	42.11 ± 6.49^c^	
21–25	40.26 ± 6.82^d^	0.033 a*
26–30	42.00 ± 2.65^cd^	
**Gender**		
Male	41.37 ± 6.78	0.635 b
Female	41.69 ± 6.53	
**Nationality**		
Emirati	41.81 ± 6.91	
Arab	41.38 ± 7.04	0.840 a
Non-Arab	41.71 ± 5.64	
**College**		
Medicine	43.94 ± 5.85^c^	
Pharmacy	36.32 ± 2.80^d^	
Dentistry	37.95 ± 5.61^d^	<0.001 a*
Nursing	45.07 ± 6.87^c^	
**Year of Study**		
1^st^	42.75 ± 6.90^c^	
2^nd^	41.83 ± 6.45^cd^	
3^rd^	40.92 ± 6.88^cd^	0.019 a*
4^th^	39.41 ± 6.03^d^	
5^th^	41.95 ± 4.51^cd^	
**Emirate of Residence**		
Ras Al Khaimah	42.81 ± 7.63	0.010 b*
Other Emirates	41.04 ± 6.04	
**Parental Education Level**		
Low	41.35 ± 7.86	0.761 b
High	41.63 ± 6.37	
**Parental Educational Background**		
Medical	40.36 ± 6.00	0.004 b*
Non-medical	42.25 ± 6.84	

^a^**P-value of ANOVA; Significant results were followed by Tukey HSD post-hoc analysis.** ^b^P- value of an independent t-test. ^c,d^Groups that do not share a superscript letter differ significantly according to Tukey HSD post-hoc analysis (p < 0.05), whereas groups sharing at least one letter are not significantly different. *P < 0.05 is statistically significant.

A higher proportion of students expressed willingness to donate organs to family members (77.7%, n = 333) compared to strangers (43.0%, n = 184) (Fig 2).

**Fig 2:**
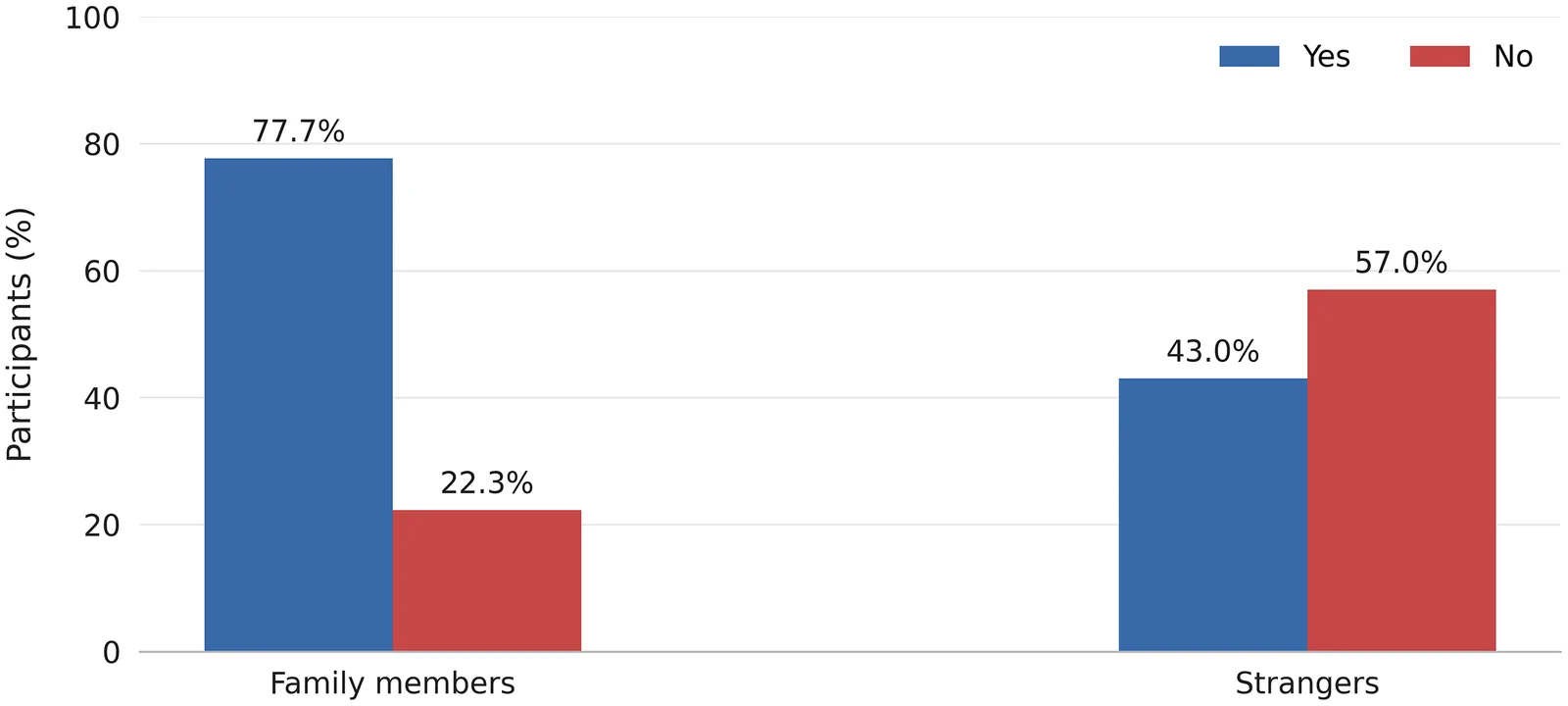
Willingness to donate organs to family members and strangers.

Only 28.0% (n = 120) of students reported being registered as organ donors, compared with 72.0% (n = 308) who were not registered. Among non-registered students (n = 308), the reported reasons were lack of awareness (39.0%, n = 120), religious concerns (26.9%, n = 83), fear of medical or surgical procedures (17.9%, n = 55), and family refusal (16.2%, n = 50) (Fig 3).

**Fig 3:**
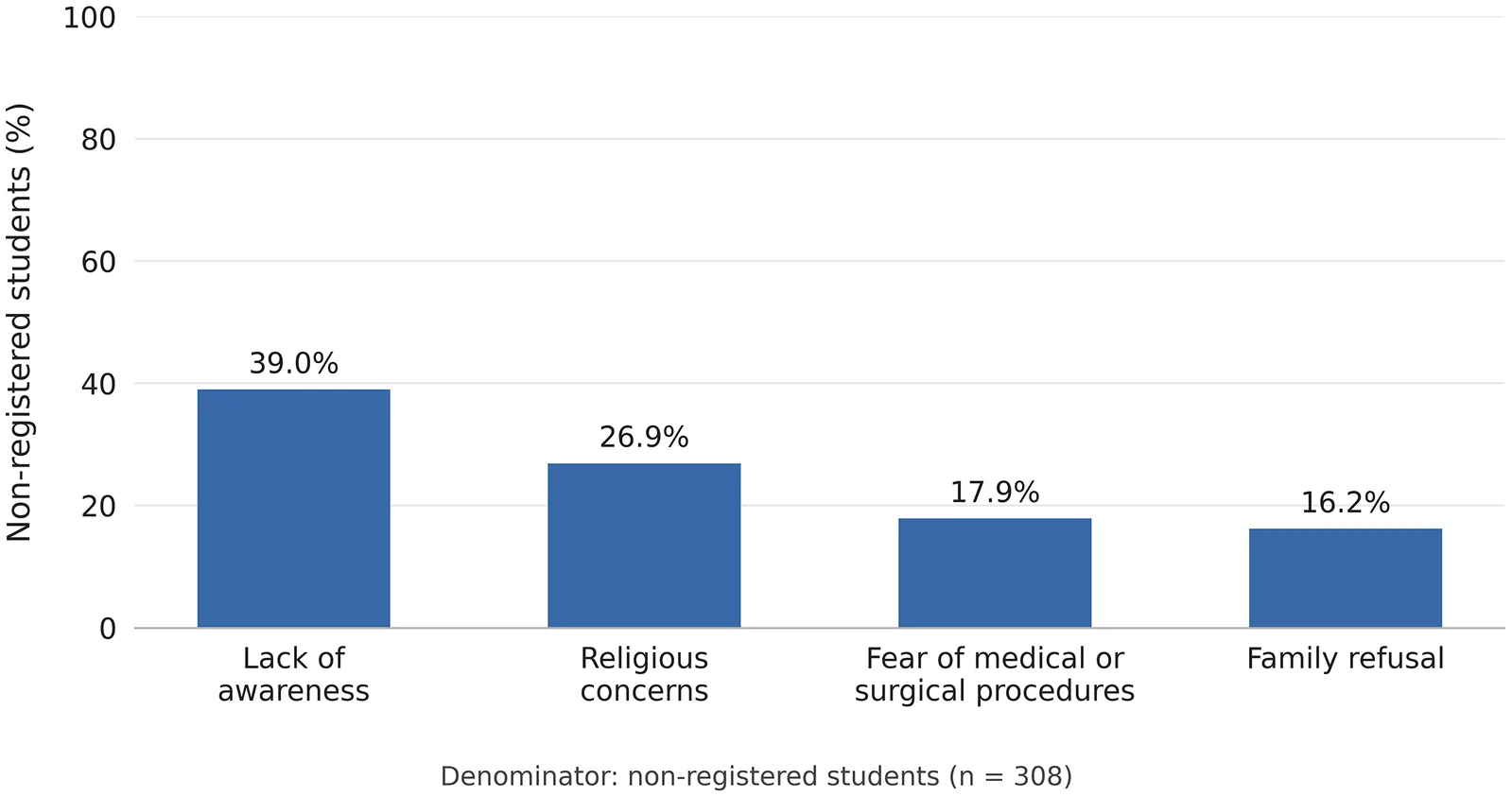
Reasons for non-registration among students who were not registered as organ donors (n = 308).

### Predictors of organ donor registration

In the multivariable binary logistic regression analysis (Table 4), students from pharmacy (aOR = 0.050, 95% CI: 0.017–0.149), dentistry (aOR = 0.032, 95% CI: 0.011–0.089), and nursing (aOR = 0.024, 95% CI: 0.008–0.067) had significantly lower adjusted odds of organ donor registration than medical students (all p < 0.001). Non-Arab students had significantly lower adjusted odds of registration than Arab students, including Emiratis (aOR = 0.149, 95% CI: 0.072–0.310, p < 0.001). Students with a medical parental educational background also had significantly lower adjusted odds of registration than those with a non-medical parental background (aOR = 0.129, 95% CI: 0.063–0.264, p < 0.001). Age, gender, year of study, parental education level, residence, knowledge score, and attitude score were not significantly associated with organ donor registration in the adjusted model.

**Table 4:** Multivariate binary logistic regression analysis of factors associated with organ donor registration (N = 428).

Variable	Category (reference)	B	aOR	95% CI	p-value
Age	Continuous	−0.071	0.932	0.752–1.154	0.517
Gender	Female vs male	0.078	1.081	0.563–2.078	0.815
Nationality	Non-Arab vs Arab incl. Emirati	−1.903	0.149	0.072–0.310	<0.001
College	Pharmacy vs Medicine	−2.996	0.050	0.017–0.149	<0.001
	Dentistry vs Medicine	−3.457	0.032	0.011–0.089	<0.001
	Nursing vs Medicine	−3.739	0.024	0.008–0.067	<0.001
Year	Year 1 vs Year 5	0.059	1.060	0.246–4.568	0.937
	Year 2 vs Year 5	−0.174	0.840	0.200–3.534	0.812
	Year 3 vs Year 5	0.663	1.941	0.471–7.991	0.359
	Year 4 vs Year 5	−0.130	0.878	0.194–3.976	0.866
Parental education	High vs low	−0.383	0.682	0.281–1.651	0.396
Parental background	Medical vs non-medical	−2.051	0.129	0.063–0.264	<0.001
Residence	Other Emirates vs RAK	−0.076	0.927	0.485–1.771	0.819
Knowledge score	Continuous	0.013	1.013	0.909–1.129	0.820
Attitude score	Continuous	−0.046	0.955	0.908–1.004	0.070

The overall model was statistically significant (likelihood-ratio χ^2^(15) = 178.38, p < 0.001). Cox and Snell R^2^ was 0.341, Nagelkerke R^2^ was 0.491, and McFadden pseudo-R^2^ was 0.351. The Hosmer–Lemeshow goodness-of-fit test did not indicate significant lack of fit (χ^2^(8) = 7.61, p = 0.473).

The model had an apparent classification accuracy of 84.1%, with a sensitivity of 56.7%, specificity of 94.8%, and an area under the ROC curve of 0.874. These performance measures represent in-sample estimates.

## Discussion

This study assessed knowledge, attitudes, and organ donor registration among medical and health sciences students in the UAE and identified key gaps between awareness, perceptions, and registration behavior. Although attitudes toward organ donation were generally favorable, knowledge remained suboptimal, particularly regarding medical, legal, and procedural aspects. Limited awareness of the national Hayat program was a key finding, suggesting that some healthcare students may be unfamiliar with the formal donor registration process. Previous research has highlighted the complex relationship between attitudes and organ donation behavior [4], while a UAE-based study by Seddiq et al. similarly identified gaps in organ donation knowledge and emphasized the need for national awareness initiatives [8]. Research among university students has also demonstrated variations in knowledge, attitudes, and willingness to donate organs [11,12]. Qualitative evidence further suggests that donation decisions are influenced by interacting informational, social, and psychological factors [13]. Although limited familiarity with registration procedures may contribute to the discrepancy between willingness and registration, this possibility requires further investigation because procedural awareness was not independently evaluated as a predictor of registration. Previous evidence on organ donation communication campaigns provides a basis for considering targeted awareness initiatives [14].

Sociodemographic and academic characteristics were associated with variations in organ donation knowledge and attitudes. Female students demonstrated significantly higher knowledge scores than male students, consistent with findings among Omani university students [5]. Gender-related differences in organ donation attitudes have also been reported in previous research [6]. Medical and nursing students also demonstrated higher knowledge scores and more favorable attitudes than dentistry and pharmacy students, potentially reflecting differences in curricular content, clinical exposure, and educational experiences across disciplines. Previous studies have similarly highlighted variations in organ donation knowledge and attitudes among healthcare students [15–17].

Variations in attitudes across age groups and academic years may further suggest that perceptions are shaped by factors beyond academic progression alone, including personal experiences and exposure to donation-related information. Differences according to emirate of residence and parental educational background may also reflect broader contextual and family-related influences. These findings suggest that educational and sociodemographic characteristics may contribute differently to knowledge and attitudes, supporting the potential value of tailored educational approaches across healthcare disciplines.

Parental educational background emerged as another relevant consideration. Higher parental education was associated with greater organ donation knowledge, suggesting a possible relationship between family educational environment and health-related awareness. However, students whose parents had medical professional backgrounds did not demonstrate significantly higher knowledge scores than those whose parents had non-medical backgrounds. Notably, parental medical background was associated with lower adjusted odds of donor registration. This unexpected finding suggests that professional familiarity with healthcare within the family does not necessarily correspond to greater participation in organ donor registration. Family communication, exposure to donation-related information, and sociocultural considerations may offer possible explanations, although these mechanisms were not directly assessed. Morgan et al. highlighted the relevance of family discussions and media exposure to organ donation decisions [18], while research among medical students in Hong Kong identified associations between family discussions, family donor registration, and individual registration [19]. Similarly, a study conducted in Ajman reported limited awareness and family opposition among barriers to organ donation [20]. Taken together, these findings suggest that family-related influences warrant further investigation, particularly in understanding the unexpected association between parental medical background and registration.

Despite generally favorable attitudes, concerns regarding body disfigurement, potential misuse of donated organs, and mistrust of healthcare systems persisted among participants. Some students also expressed concern that donor registration might compromise the quality or priority of emergency medical care. Such perceptions may represent psychological and trust-related barriers that are not necessarily resolved by general knowledge of organ donation. Previous research has identified personal beliefs and concerns as relevant to willingness to donate [21], while other studies have highlighted the relationship between knowledge, religious beliefs, and donation attitudes [22]. Communication and other potentially modifiable factors have also been examined in relation to family donation decisions [23]. These findings emphasize the importance of addressing misconceptions and concerns alongside factual knowledge, although the independent contribution of these perceptions to registration was not established in the present study.

An important finding was the discrepancy between participants’ stated willingness to donate organs and their self-reported registration status. This suggests that favorable attitudes and willingness do not necessarily correspond to formal donor registration. The finding is consistent with the intention–behavior gap described in behavioral research [24] and with evidence examining associations between attitudes and organ donation behaviors [25]. Psychological, social, and contextual factors may contribute to this discrepancy, as suggested by theory-based research on donation intentions [26]. The Theory of Planned Behavior offers a useful conceptual framework for interpreting these findings, particularly through the potential roles of subjective norms and perceived behavioral control [27,28]. However, these constructs were not directly measured in the present study, and their contribution to donor registration warrants further investigation.

Within the UAE's sociocultural context, family expectations, religious considerations, and trust in healthcare institutions may influence perceptions of organ donation and individual decision-making. Previous research has identified associations between knowledge, attitudes, and willingness to donate organs [29], while studies among medical students have examined the relationship between donation-related perceptions and behaviors [30]. The relatively low registration rate observed in the present study, despite generally favorable attitudes, may therefore reflect a combination of informational, psychological, and contextual influences. Research among university students in the UAE has also examined organ donation attitudes within the local sociocultural environment [31], while broader literature has emphasized the relevance of religious considerations and faith-based engagement in donation decisions [32].

Willingness to donate organs was also reported more frequently for family members than for strangers, suggesting that familial relationships may influence donation preferences. Religious and cultural considerations may further shape these preferences, particularly regarding the permissibility of donation and beliefs surrounding death and bodily integrity. Previous literature has discussed Islamic perspectives on organ donation and transplantation, including the ethical and religious considerations involved [33,34]. These findings suggest that culturally sensitive communication, supported by healthcare professionals, community organizations, and religious authorities, may help address misconceptions and encourage informed decision-making.

The findings also have implications for organ donation education and awareness initiatives. A recent systematic review of interventions among university students identified educational programs, peer-led activities, and digital communication as potential approaches to improving donation-related knowledge and attitudes [35]. Social-media platforms may also provide opportunities to connect individuals with official donor registration systems, as illustrated by increased online registrations observed following a social-media initiative [36]. Nevertheless, a Cochrane systematic review indicates that evidence regarding the effectiveness of interventions in increasing formal organ donor registration remains variable [37]. Evidence from a randomized controlled trial among nursing students further supports the potential value of structured educational interventions in healthcare education [38]. In the UAE, integrating organ donation education with accessible, accurate information about the Hayat program may therefore represent a practical approach for future evaluation. Such initiatives should address not only knowledge gaps but also procedural uncertainty, misconceptions, and family-related concerns, with formal registration assessed as an outcome rather than assuming that improved attitudes will necessarily translate into action.

### Study limitations

Some limitations should be considered when interpreting the findings. First, the cross-sectional design precludes causal inference and does not establish a temporal relationship between willingness and donor registration. Moreover, subjective norms and perceived behavioral control were not directly assessed; therefore, the Theory of Planned Behavior was used only as a conceptual framework rather than being formally tested. Second, reliance on self-reported data may have introduced recall and social desirability biases, potentially affecting the accuracy of reported knowledge, attitudes, and registration status. Third, consecutive non-probability sampling may have introduced selection bias, while the single-institution setting limits the generalizability of the findings to health sciences students at other universities and across the UAE. Additionally, unmeasured factors, including family communication, trust in healthcare institutions, and familiarity with registration procedures, may have influenced the observed associations. Future multicenter studies using representative sampling and mixed-methods approaches are warranted to validate these findings and provide a more comprehensive understanding of the barriers to organ donor registration.

### Recommendations

Based on the study findings, universities should consider integrating structured education on organ donation into health sciences curricula, covering clinical, ethical, legal, and religious aspects. Collaboration with the Ministry of Health and Prevention could enhance awareness of the national Hayat program through campus-based educational initiatives and improved access to registration information, including QR codes linking to the official registration platform. Social media campaigns and peer-led educational activities may also help address misconceptions and improve awareness of organ donation procedures. Furthermore, culturally sensitive engagement involving families, healthcare professionals, and religious scholars may help address concerns, strengthen trust, and support informed decision-making. Future studies should evaluate the effectiveness of these strategies in improving knowledge, attitudes, and formal donor registration.

## Conclusions

The findings identified gaps in organ donation knowledge, particularly regarding the national Hayat program, despite generally positive attitudes toward organ donation. However, concerns regarding organ misuse, body disfigurement, lack of trust, and the perceived risk of reduced quality of medical care remained. Only 28.0% of participants reported being registered as organ donors, highlighting a gap between reported attitudes and actual registration. Nationality, college, and parental medical background were significantly associated with donor registration. These findings support consideration of targeted educational initiatives that address knowledge gaps, clarify registration procedures, and respond to students’ concerns. Further multicenter studies are warranted to validate these associations and explore barriers to registration, particularly procedural awareness, family communication, and trust in the organ donation system.
